# Omental adipose tissue is a more suitable source of canine Mesenchymal stem cells

**DOI:** 10.1186/s12917-017-1053-0

**Published:** 2017-06-08

**Authors:** Francisca Bahamondes, Estefania Flores, Gino Cattaneo, Flavia Bruna, Paulette Conget

**Affiliations:** 1 Centro de Medicina Regenerativa, Facultad de Medicina, Clínica Alemana - Universidad del Desarrollo, Av. Las Condes 12,438, Lo Barnechea, Santiago, 7710162 Chile; 2 Departamento de Ciencias Clínicas, Facultad de Ciencias Veterinarias y Pecuarias, Universidad de Chile, Av. Santa Rosa 11,735, Santiago, Chile

**Keywords:** Mesenchymal stem cell, Source, Adipose tissue, Omentum, Canine, Dog

## Abstract

**Background:**

Mesenchymal Stem Cells (MSCs) are a promising therapeutic tool in veterinary medicine. Currently the subcutaneous adipose tissue is the leading source of MSCs in dogs. MSCs derived from distinct fat depots have shown dissimilarities in their accessibility and therapeutic potential. The aims of our work were to determine the suitability of omental adipose tissue as a source of MSCs, according to sampling success, cell yield and paracrine properties of isolated cells, and compared to subcutaneous adipose tissue.

**Results:**

While sampling success of omental adipose tissue was 100% (14 collections from14 donors) for subcutaneous adipose tissue it was 71% (10 collections from 14 donors). MSCs could be isolated from both sources. Cell yield was significantly higher for omental than for subcutaneous adipose tissue (38 ± 1 vs. 30 ± 1 CFU-F/g tissue, *p* < 0.0001). No differences were observed between sources regarding cell proliferation potential (73 ± 1 vs. 74 ± 1 CDPL) and cell senescence (at passage 10, both cultures presented enlarged cells with cytoplasmic vacuoles and cellular debris). Omental- and subcutaneous-derived MSCs expressed at the same level bFGF, PDGF, HGF, VEGF, ANG1 and IL-10. Irrespective of the source, isolated MSCs induced proliferation, migration and vascularization of target cells, and inhibited the activation of T lymphocytes.

**Conclusion:**

Compared to subcutaneous adipose tissue, omental adipose tissue is a more suitable source of MSCs in dogs. Since it can be procured from donors with any body condition, its collection procedure is always feasible, its cell yield is high and the MSCs isolated from it have desirable differentiation and paracrine potentials.

**Electronic supplementary material:**

The online version of this article (doi:10.1186/s12917-017-1053-0) contains supplementary material, which is available to authorized users.

## Background

Mesenchymal stem cells (MSCs) are non-hematopoietic precursor cells that can be differentiated, among others, into chondrocytes, osteocytes and adipocytes [1]. Together, MSCs secrete trophic, vasculogenic and immunomodulatory factors that have a paracrine effect on tissue resident cells [2–4]. Hence, MSCs are a promissory therapeutic tool for regenerative medicine [5].

The leading source of MSCs is the bone marrow. In 2001, they were isolated for the first time from adipose tissue [6]. When compared to bone marrow, adipose tissue appeared as a superior source of MSCs due to the fact that a less invasive procedure is required to procure it [7–9].

In veterinary medicine, it has been proven that the administration of adipose-derived MSCs have therapeutic effects in small animal patients, particularly in cats and dogs [10–13]. Adult dogs have adipose tissue locates mainly in subcutaneous and visceral depots. Thus, adipose tissue may be procured through minimally invasive procedures from arms, thighs and abdomen (subcutaneous), or from omentum, kidney and liver (visceral) [14, 15]. While the abundance of subcutaneous adipose tissue depends on the body condition, the extent of omentum is relatively constant [16, 17]. It has been demonstrated that there are MSCs in the omentum of both dogs [10, 18] and humans [19, 20]. Omental-derived MSCs are similar to subcutaneous-derived MSCs according to their proliferation and surface antigen expression [18]. Up to our knowledge, no data are available regarding the abundance and paracrine potential of omental-derived MSCs. Since previous studies showed that adipose-derived MSC properties vary from depot to depot [21], it will be also interesting to compare MSCs isolated form omentum with those isolated from subcutaneous fat, the leading adipose source of MSCs.

The aims of our work were to determine the suitability of omental adipose tissue as a source of MSCs, according to sampling success, cell yield and paracrine properties of isolated cells, and compared to subcutaneous adipose tissue.

## Methods

### Collection of adipose tissue

Fourteen female dogs of different breeds, 6–12 months old, with normal corporal condition, clinically healthy and having elective ovariohysterectomies were enrolled in the study, after client-owned provided written informed consent. Animals were pre-anesthetized with 0.04 mg/Kg Acepromazine (Holliday*-*Scott S.A, Buenos Aires, Argentina). General anesthesia was induced with 3 mg/Kg Propofol (Fresenius Kabi, Spain) and maintained with Isofluorane (Baxter Healthcare Corporation*,* Deerfield, IL). Approximately 3cm^3^ (5-10 g) of omental and subcutaneous adipose tissue were procured from greater omentum and abdominal region, respectively. Subsequently, an ovariohysterectomy was performed. Muscular and skin incisions were sutured with a simple, interrupted pattern. Up to three days post-surgery animals received 1 mg/Kg/24 h Ketoprofen (Merial Laboratorios, Argentina).

Study was approved by Ethic Committee Facultad de Ciencias Veterinarias y Pecuarias, Universidad de Chile (No. 03–2014).

### Isolation, ex vivo expansion and characterization of MSCs

Adipose tissue samples were weighed, washed with phosphate buffered saline (PBS. Sigma, St. Louis, MO, USA) containing 80 μg/mL gentamycin (Sanderson Laboratory, Santiago, Chile), minced with scissors and scalpels, and digested in PBS containing 1 mg/mL collagenase type II (Gibco, Grand Island, NY, USA), at 37 °C, overnight. Enzyme activity was neutralized with alpha-MEM (Gibco, Auckland, NZ) supplemented with 10% fetal bovine serum (Gibco, Auckland, NZ) and 80 μg/mL gentamicin (Sanderson Laboratory, Santiago, Chile) (here after expansion medium), and centrifuged at 400×g for 10 min. Pelleted cells were resuspended in expansion medium and plated at a density of 50,000 nucleated cells/cm^2^ and cultured under an atmosphere with 5% CO_2_, at 37 °C. Fourty eight hours later, nonadherent cells were removed by media change. When 80% confluence was achieved, adherent cells were detached with 0.25% trypsin and 2.65 mM EDTA, centrifuged and subcultured at 5000 cells/cm^2^. After two subcultures, adherent cells were characterized according to their adipogenic [21], chondrogenic [22] and osteogenic differentiation potential [23]. Although there are currently no consensus markers for canine MSCs as there are for human MSCs [1], immunophenotyping was performed by flow cytometry analysis after labeling with monoclonal antibodies against: CD45^FITC^, CD11b^PE-Cy5^, CD44^APC^ and CD90^PE^ or their respective isotype controls (rat IgG2b^FITC^, rat IgG2b^PE-Cy5^, rat IgG2b^APC^ or rat IgG2b^PE^; eBioscience, San Diego, CA).

### Fibroblast-like Colony forming unit (CFU-F) assay

CFU-F assay was performed on freshly isolated cells as previously described [24]. Briefly, 500 mononuclear cells/cm^2^ were cultured in expansion medium. At day 7, cells were fixed with 4% paraformaldehyde for 10 min and stained with 0.5% crystal violet (Sigma-Aldrich, St. Louis, MO) in 10% methanol for 20 min. Plates were observed under light microscope (Leica DM2000). Clusters containing more than 50 cells were scored as CFU-Fs and counted. Results were expressed as CFU-F per gram of tissue (CFU-F/g tissue).

Assays were performed in triplicate.

### Evaluation of cumulative population doubling level (CPDL) and senescence

One thousand cells/cm^2^ were seeded and cultured with expansion medium. The medium was changed every three days and cells were subcultured when reaching 80% confluence. The population doubling (PD) at each subculture was calculated according to the formula PD = ln (*N*_*f*_ */N*_*i*_) /ln 2, where *N*_*i*_ and *N*_*f*_ are initial and final cell numbers, respectively. The PDs of continuous subcultures were added to obtain CPDL [10].

Senescence was assessed looking for changes in cell morphology such as cell enlargement, accumulation of vacuoles and presence of cellular debris [25].

Assays were performed in triplicate.

### RT-qPCR

RNA was extracted from cells using Tryzol (*Invitrogen*, Carlsbad, CA, USA) and treated with DNAse (*Invitrogen*, Carlsbad, CA, USA) following the manufacturer’s instructions. One μg of RNA was reverse-transcribed using oligo-dT primers and Moloney murine leukemia virus reverse transcriptase. The abundance of mRNA was determined by qPCR using SYBR Green Technology and canine-specific primers for bFGF, PDGF HGF, VEGF, ANG1, IDO, IL-10 and 18S (Additional file 1: Table S1). Cycling condition were: 1 cycle, 94 °C for 10 min; 30–35 cycles, 94 °C for 10 min; optimal annealing temperature for 5 min; 72 °C for 4 min; 1 cycle, 64 °C for 10 min; 1 cycle, 40 °C for 30 min. The qPCR products were separated by electrophoresis on 2% agarose gel, stained with 1% ethidium bromide and visualized under UV light. Digital images were captured with Alpha imagen software. Values were normalized to 18S mRNA levels. Relative gene expression was quantified with the 2 ^-ΔΔCt^ method [26].

### Proliferation assay

Human fibroblasts were seeded at 4000 cells/cm^2^ and cultivated with alpha-MEM (control) or alpha-MEM conditioned by MSCs for 24 h. The medium was changed every 3 days. Three, six, nine and 12 days later, cells were stained with 0.5% crystal violet in 10% methanol for 20 min. After four washes, crystal violet incorporated into the cells were solubilized with 50% methanol in PBS and quantified spectrophotometrically (absorbance at 570 nm) [24].

Assays were performed in triplicate.

### Scratch assay

Human fibroblasts were seeded at 8000 cells/cm^2^. After 24 h, a line in the monolayer was performed with a sterile p200 pipette tip and medium was changed by alpha-MEM (control) or alpha-MEM conditioned by MSCs for 24 h. Zero, six, and 12 h after scratching images were captured under a light microscope (Leica DM2000) with a digital camera (Leica DFC 295). Image J software (http://rsbweb.nih.gov/ij/) was used to quantify the scratch area [27].

Assays were performed in triplicate.

### Tube formation assay

Human umbilical vein endothelial cells (HUVECs) were seeded at 3000 cells/cm^2^ on 10 mg/mL growth factor-reduced Matrigel (BD Biosciences, Boston, MA, USA) and exposed to alpha-MEM (control), alpha-MEM conditioned by MSCs for 24 h or endothelial growth medium (Lonza, Walkersville, MD, USA) (positive control) [28]. Five hours later, images were captured under a light microscope using a digital camera. WimTube program (Wimasis GmbH, Munich, Germany) was used to quantify i) total tube length, ii) total branching point, iii) total loops.

Assays were performed in triplicate.

### T lymphocyte proliferation assay

Canine peripheral blood mononuclear cells were labeled with carboxyfluorescein succinimidyl ester (Invitrogen/Molecular Probes, Eugene, OR) and cultivated with alpha-MEM (control) or alpha-MEM conditioned by MSCs for 24 h, supplemented with 4 uL (1:10) phytohaemaglutinin (Gibco, Invitrogen, Corporation, Scotland, UK). Five days later, cells were stained with monoclonal antibody against CD4^PE^ (Serotec, Kidlington, Oxford, United Kingdom) and analyzed by flow cytometry [29].

Assays were performed in triplicate.

### Statistical analysis

Data are presented as mean ± S.E.M. To determine the statistical significance of intergroup differences a one-way ANOVA test was used to compare mean values among all groups and Student’s unpaired *t*-test or Mann-Whitney test (non parametric) was used to compare mean values between two groups. *p* < 0.05 was considered as statistically significant.

## Results

### Omental adipose tissue has higher sampling success than subcutaneous adipose tissue

The age and weight of donor dogs were 10 ± 3 months and 12 ± 6 Kg (Table 1). Omental samples were procured from the 14 donors and subcutaneous samples were procured from 10 of them. Thus, the success sampling rates were 100% (14 of 14) for omental adipose tissue and 71% (10 of 14) for subcutaneous adipose tissue (Table 1). The average weight of the procured samples was 5.2 ± 4.5 g for omental and 2.4 ± 3.4 g for subcutaneal adipose tissue (Table 1).

**Table 1:** Enrolled animals and procured samples characteristics

Donor (identifier)	Age (months)	Weight (kilograms)	Subcutaneous (grams)	Omental (grams)
B001	8	8	not available	6.3
P002	12	15	4.8	8.1
L003	7	11	4.6	9.8
P004	8	12	not available	3.1
K005	12	15	1.9	2.3
L006	7	12	4.5	8.4
P007	8	10	3.2	4.5
C008	12	16	3.1	7.1
C009	11	6	not available	3.2
S010	8	15	5.8	5.9
O011	8	7	not available	3.7
C012	12	10	1.5	3.0
P013	10	6	2.3	4.8
S014	10	18	3.3	3.2
TOTAL	10 ± 3	12 ± 6	2.4 ± 3.4	5.2 ± 4.5

Further characterization of adipose-derived MSCs was performed for samples obtained from the 10 donors in whom it was possible to obtain both omental and subcutaneous adipose tissues**.**

### Omental adipose tissue and subcutaneous adipose tissue have MSCs

**Fig. 1:**
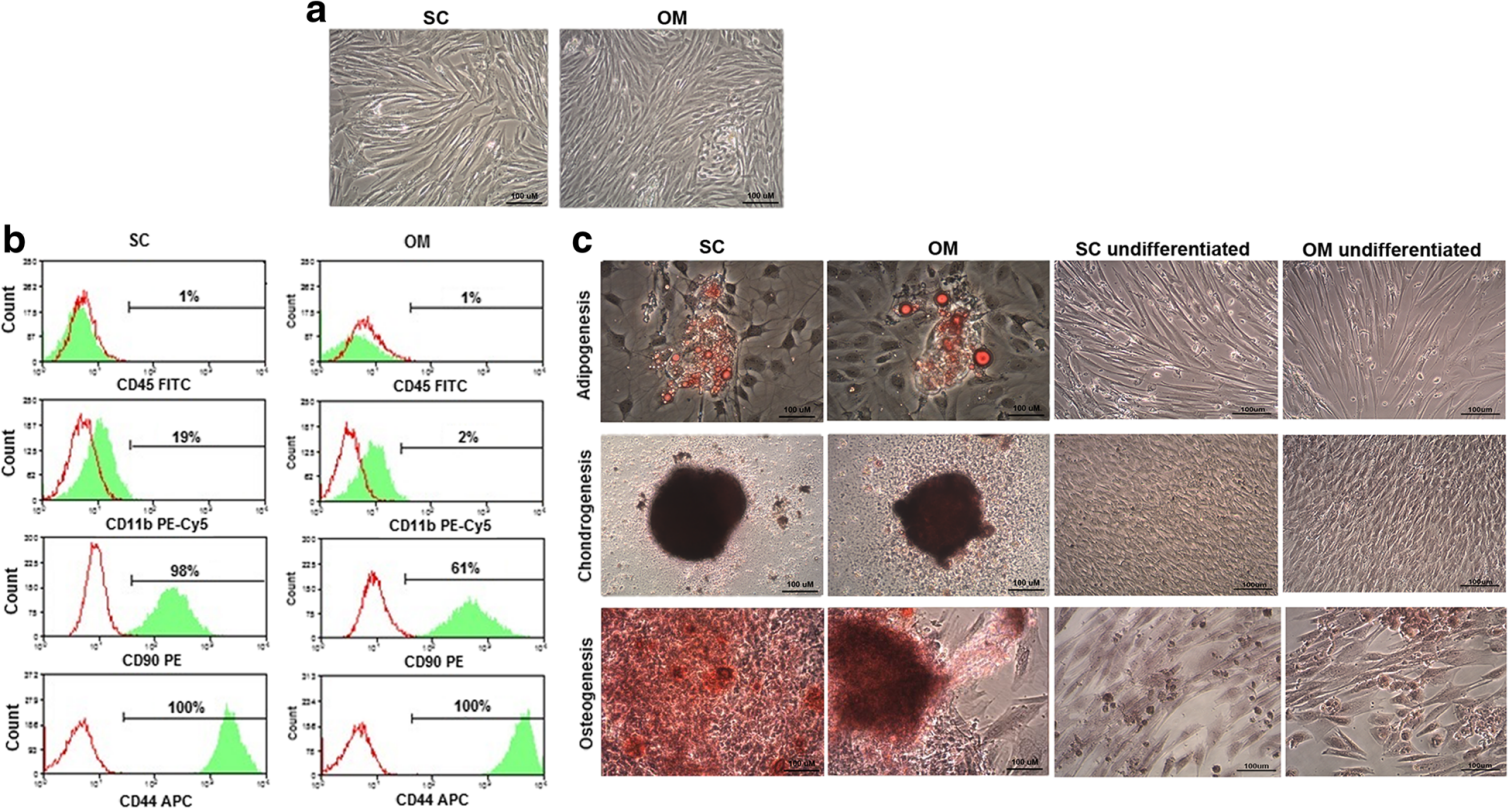
Omental adipose tissue and subcutaneous adipose tissue have MSCs. **a** Representative photomicrographs of primary culture of subcutaneous- and omental-derived cells. **b** Flow cytometry analysis after immunostaining with monoclonal antibodies against CD45, CD11b, CD90 and CD44 (*green lines*) or isotype controls (*red lines*). **c** Representative photomicrographs of MSCs stained with Oil Red O (adipogenesis), Safranin O (chondrogenesis) or Alizarin Red (osteogenesis) 3 weeks after differentiation induction. Control undifferentiated cells are also shown. Abbreviations: SC: subcutaneous adipose tissue; OM: omental adipose tissue

Cells isolated from both sources adhered to plastic and showed fibroblast-like morphology (Fig. 1a). Together, they were negative for hematopoietic markers (CD45 and CD11b) and positive for MSC markers (CD90 and CD44) (Fig. 1b). When exposed to adipogenic stimulus, cells differentiated into adipocytes that accumulate lipid droplets throughout the cytoplasm as confirmed by Oil Red O staining (Fig. 1c). After 21 days under chondrogenic induction, sulfated glycosaminoglycans were present in the matrix as revealed by Safranin O staining (Fig. 1c). Cell osteogenic differentiation was confirmed due to the appearance, 3 weeks after exposure to osteogenic medium, of calcium deposits that stained with Alizarin Red (Fig. 1c).

### Omental adipose tissue has higher abundance of MSCs than subcutaneous adipose tissue

**Fig. 2:**
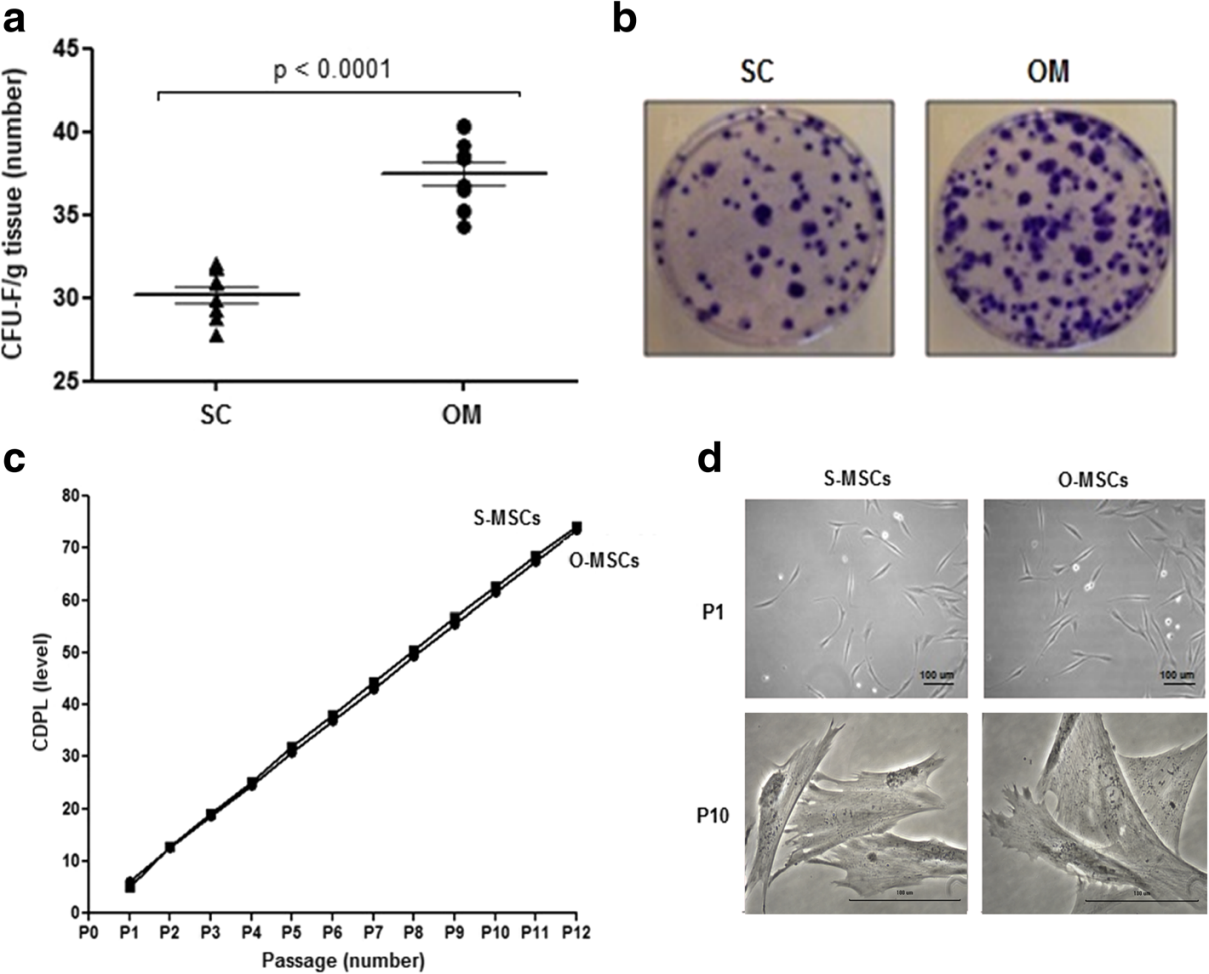
Omental adipose tissue has higher abundance of MSCs than subcutaneous adipose tissue. **a** Quantitative analysis of CFU-Fs per gram of tissue sampled. **b** Representative photographs of plates stained with crystal violet 7 days after seeding of nucleated cells from tissue samples. **c** Quantitative analysis of CPDL up to passage 12. **d** Representative photomicrographs of S-MSCs and O-MSCs at passages 1 (P1) and 10 (P10). Abbreviations: SC: subcutaneous adipose tissue; OM: omental adipose tissue; CFU-F/g tissue: colony forming units per grams of tissue; S-MSCs: mesenchymal stem cells derived from subcutaneous adipose tissue; O-MSCs: mesenchymal stem cells derived from omental adipose tissue; CDPL: cumulative population doubling level

The relative abundance of MSCs in omental adipose tissue was 38 ± 1 CFU-F/g tissue and in subcutaneous adipose tissue was 30 ± 1 CFU-F/g tissue, *p* < 0.0001 (Fig. 2a-2b).

### MSCs derived from omental and subcutaneous adipose tissues have a similar expansion potential

No statistical difference was observed bewteen omental- and subcutaneous-derived MSCs regarding their proliferation potential up to passage 12 (73 ± 1 vs. 74 ± 1 CDPL) (Fig. 2c). Senescence characteristics such as cell enlargement, generation of vacuoles and presence of cellular debris were seen from passage 10 in both MSCs (Fig. 2d).

### MSCs derived from omental and subcutaneous adipose tissues have similar trophic properties

**Fig. 3:**
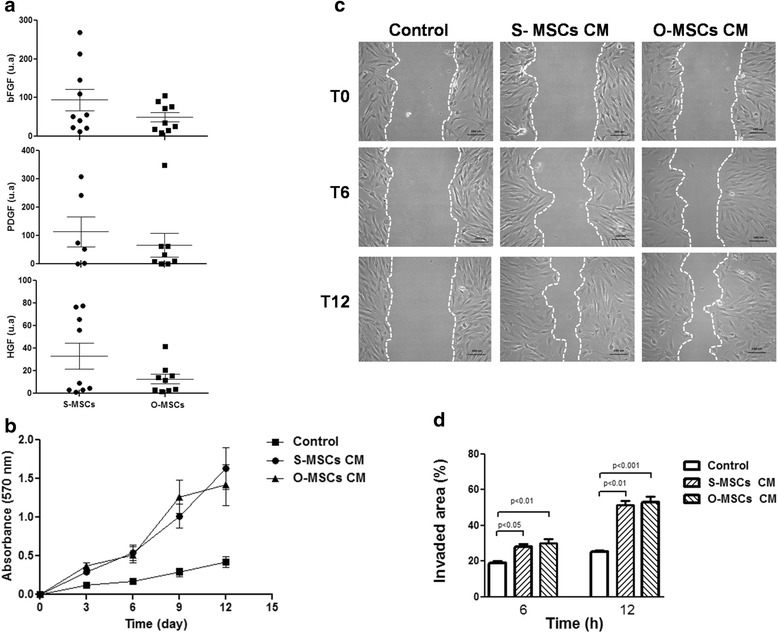
MSCs derived from omental and subcutaneous adipose tissues have similar trophic properties. **a** Quantitative analysis of mRNA levels of trophic factors. **b** Quantitative analysis of proliferation kinetic of human fibroblast cultivated with S-MSCs CM or O-MSCs CM evaluated during 12 days. **c** Representative photomicrographs of invaded area at 0 (T0), six (T6) and 12 (T12) hours post exposure to CMs. **d** Quantitative analysis of invaded area evaluated as the percentage of area free of cells at 6 or 12 h post exposure to CM with respect to 0 h. Abbreviations: S-MSCs: mesenchymal stem cells derived from subcutaneous adipose tissue; O-MSCs: mesenchymal stem cells derived from omental adipose tissue; CM: conditioned medium; **bFGF**: basic fibroblast growth factor; **PDGF**: platelet-derived growth factor; **HGF**: hepatocyte growth factor

Both adipose-derived MSCs expressed at the same level the trophic factors bFGF, PDGF and HGF (Fig. 3a). Accordingly, no differences were observed in their potential to promote human fibroblast proliferation and migration (Fig. 3b, c and d).

### MSCs derived from omental and subcutaneous adipose tissues have similar vasculogenic properties

**Fig. 4:**
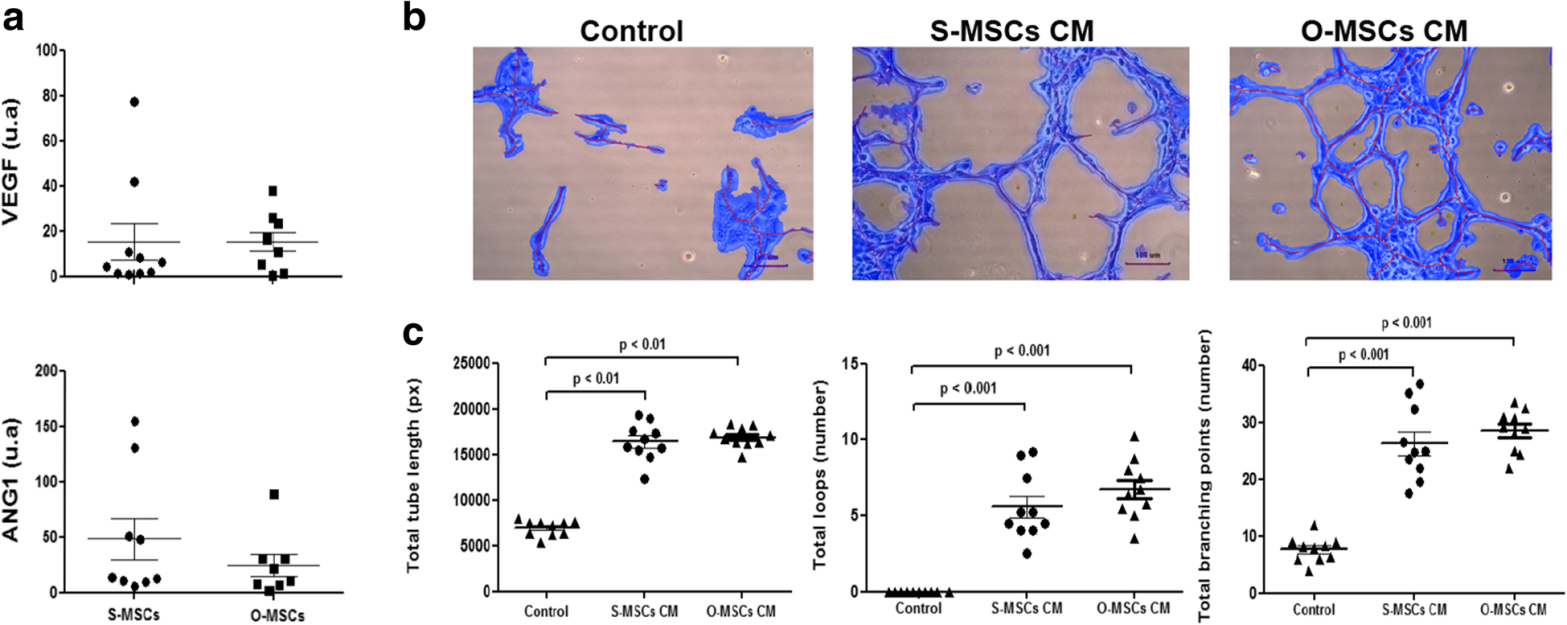
MSCs derived from omental and subcutaneous adipose tissues have similar vasculogenic properties. **a** Quantitative analysis of mRNAs levels of vasculogenic factors. **b** Representative photomicrographs of tube assay performed with 3D collagen-embedded human umbilical endothelial cells cultivated 5 h with S-MSCs CM or O-MSCs CM and analyzed by Wimasis software. **c** Quantitative analysis of total tube length, total branching point and total loops of tube assay among the different conditions. Abbreviations: S-MSCs: mesenchymal stem cells derived from subcutaneous adipose tissue; O-MSCs: mesenchymal stem cells derived from omental adipose tissue; CM: conditioned medium; **VEGF**: vascular endothelial growth factor; **ANG1** angiopoietin 1

Irrespective of their origin, adipose-derived MSCs expressed VEGF and ANG1 (Fig. 4a). Consequently, they equally promote tube formation of HUVEC (total tube length, total branching point and total loops) (Fig. 4b and c).

### MSCs derived from omental and subcutaneous adipose tissues have similar immunomodulatory properties

**Fig. 5:**
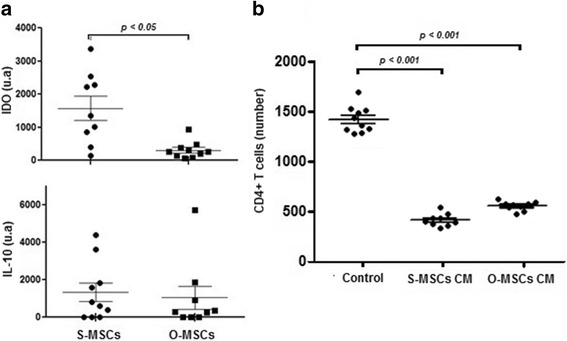
MSCs derived from omental and subcutaneous adipose tissues have similar immunomodulatory properties. **a** Quantitative analysis of the mRNAs levels of immunomodulatory factors. Each value was normalized to 18S expression. **b** Quantitative analysis of total CD4+ T-cells stimulated and cultivated with S-MSC CM and O-MSC CM after 5 days. Abbreviations: S-MSCs: mesenchymal stem cells derived from subcutaneous adipose tissue; O-MSCs: mesenchymal stem cells derived from omental adipose tissue; CM: conditioned medium; **IDO** indoleamine-pyrrole 2,3-dioxygenase; **IL-10** interleukin 10

The gene expression level of IDO was significantly lower in omental MSCs than in subcutaneous cells (*p* < 0.05. Fig. 5a). The level of IL-10 did not differ significantly between them. Both adipose-derived MSCs prevent CD4+ T cell-proliferation at the same extent (Fig. 5b).

## Discussion

A major challenge associated with MSC-based therapies is the selection of the source [14]. Here we showed in young and healthy female dogs that, compared to subcutaneous adipose tissue, omental adipose tissue is a more suitable source of MSCs. Since it can be procured from donors with any body condition, its collection procedure is always feasible, its cell yield is high and the MSCs isolated from it have desirable differentiation and paracrine potentials.

The fact that omental but not subcutaneous adipose tissue was always procurable may be attributed to volume variability of fat depots as well as to the expertise of the professional that procures them. In a study with 1265 dogs, it was shown that the size of collected samples was determined by the location of the adipose tissue, being visceral samples bigger than subcutaneous ones [12]. This difference might be critical when donor should be an emaciated patient due to either a chronic or a nutritional disease.

We showed that omental adipose tissue yielded a higher number of viable MSCs per gram of tissue than subcutaneous adipose tissue. These results are in agreement with other studies showing the same differences in humans [14] and dogs [10, 15].

Consistent with the results reported for human MSCs, canine MSCs isolated either from omental or subcutaneous adipose tissue followed a lineal trend of proliferation up to passage 12 and senescence characteristics appeared at passage 10 [23, 30]. Thus, our data support a significant but limited expansion potential of canine MSCs. Hence, the feasibility to be procured and the cell richness of the sample take higher relevance in order to choose the best source of canine MSCs.

The secretory activity of MSCs favors a regenerative microenvironment [31, 32]. The trophic properties of MSCs depend on the secretion of growth factors that induce cell proliferation and migration [33–35]. We showed that canine MSCs isolated from omental or subcutaneous tissues express bFGF, PDGF and HGF. Both bFGF and PDGF elicit target cell proliferation after ligand-binding induction of receptor oligomerization, activation of intrinsic receptor tyrosine kinase and phosphorylation of specific residues in the cytoplasmic region [36–39]. HGF is a pleiotropic factor displaying mitogenic, motogenic, morphogenetic, and antiapoptotic activities in different target cells [40]. Indeed, HGF promotes hepatocytes, keratinocytes, renal tubule cell and endothelial cell proliferation, dissociation of epithelial cell colonies, cell motility, and invasion through extracellular matrix. More recently, it has been shown that bFGF, PDGF and HGF can induce biological responses also on MSCs [41–43]. Our functional studies showed that, irrespective of the source, canine MSCs secrete active mitogenic and motogenic factors.

Vasculogenesis is a crucial step in the wound healing process [44, 45]. The formation of new blood vessels is necessary to sustain the newly formed granulation tissue and the survival of keratinocytes. In this study, we found that adipose tissue derived MSCs express VEGF and ANG1. Both stimulate endothelial cell proliferation, migration, and organization into tubules [46, 47]. Our functional study showed that MSCs either form omental or subcutaneous tissue secreted active factors that promote vasculogenesis.

Since in the functional assays we used human fibroblasts or human endothelial cells as target cells, our data prove than trophic and vasculogenic factors secreted by MSCs isolated from dog omental or subcutaneous adipose tissue overcome species-specificity barrier. In order to further characterize the products secreted by canine MSCs it would be relevant to perform the functional experiments using target cells from dogs and other species.

Much attention has been paid to the immunomodulatory properties of MSCs. Several studies have shown a paracrine suppressive effect on T cells, B cells, monocytes and macrophages [48–50]. We showed similar gene expression levels of IL-10 in MSCs from both sources studied. Though, the expression of IDO in MSCs derived from subcutaneous adipose tissue was 11-fold higher than in MSCs derived from omentum. IDO catalyzes the conversion of tryptophan to kynurenine and inhibits T cell proliferation due to tryptophan depletion [51]. Nevertheless, this is not the unique mechanism supporting the immunosuppresive potential of MSCs [52–55]. That should explain why, despite of the differences observed in IDO mRNA levels, in the functional assay MSCs from both sources inhibit at the same magnitude CD4+ T cell proliferation. Our results appear consistent with previous findings for human and canine MSCs [4, 56, 57].

Data here presented not only shall be useful for evidence based-selection of MSC source but also to expand the frontiers of the use of canine MSCs as they prove to produce active trophic, vasculogenic and immunomodulator soluble factors.

## Conclusion

Compared to subcutaneous adipose tissue, omental adipose tissue is a more suitable source of MSCs in dogs. Since it can be procured from donors with any body condition, its collection procedure is always feasible, its cell yield is high and the MSCs isolated from it have desirable differentiation and paracrine potentials.

## Additional file

Additional file 1: Table S1. Genes, primers and amplicon characteristics. (TIFF 552 kb)

## Abbreviations

ANG1: Angiopoietin 1
bFGF: Basic fibroblast growth factor
CDPL: Cumulative population doubling level
CFU-F: Fibroblastic-like colony forming unit
CM: Conditioned medium
EDTA: Ethylenediaminetetraacetic acid
HGF: Hepatocyte growth factor
HUVEC: Human umbilical vein endothelial cells
IDO: Indoleamine-pyrrole 2,3-dioxygenase
IL-10: Interleukin 10
MSCs: Mesenchymal stem cells
OM: Omental adipose tissue
O-MSCs: Omental mesenchymal stem cells
PBS: Phosphate-buffered saline
PDGF: Platelet-derived growth factor
SC: Subcutaneous adipose tissue
S-MSCs: Subcutaneous mesenchymal stem cells
VEGF: Vascular endothelial growth factor
