# Supplementary figures and images for: Omental adipose tissue is a more suitable source of canine Mesenchymal stem cells
Source: BMC Vet Res. 2017 Jun 8;13:166. doi: 10.1186/s12917-017-1053-0 (PMC5465460; doi:10.1186/s12917-017-1053-0)

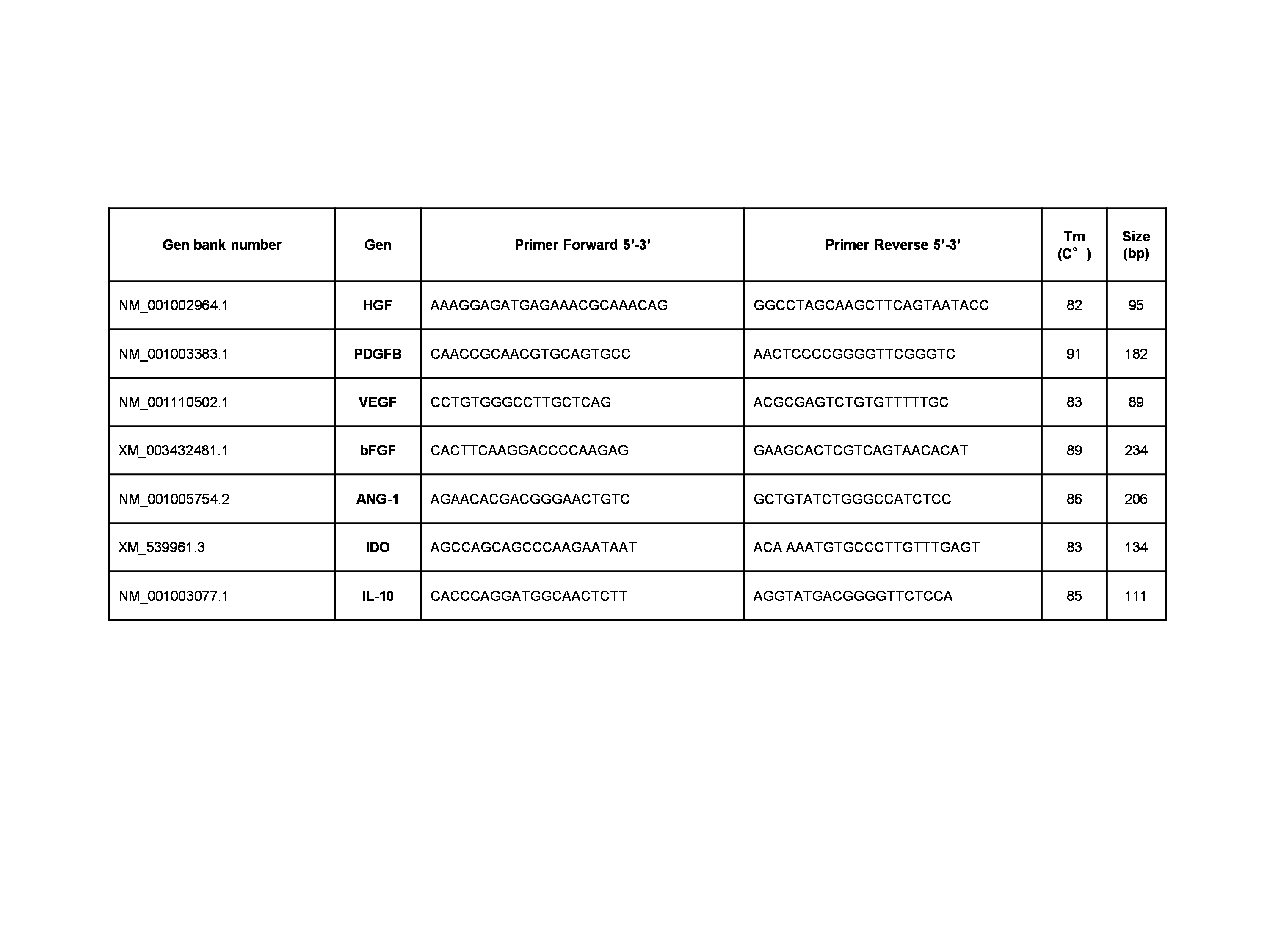

Supplement: Additional file 1: Table S1. — Genes, primers and amplicon characteristics. (TIFF 552 kb) [file 12917_2017_1053_MOESM1_ESM.tif]
